# Balancing act: progress and prospects in breeding soybean varieties with high oil and seed protein content

**DOI:** 10.3389/fpls.2025.1560845

**Published:** 2025-10-21

**Authors:** Qingchi Shi, Weiliang Mo, Xunan Zheng, Xuelai Zhao, Xiaoyu Chen, Li Zhang, Jianchun Qin, Zhenming Yang, Zecheng Zuo

**Affiliations:** ^1^ Jilin Province Engineering Laboratory of Plant Genetic Improvement, College of Plant Science, Jilin University, Changchun,, China; ^2^ College of Animal Science, Guangxi University, Nanning, China

**Keywords:** Soybean (Glycine max), seed oil content, seed protein content, trade-off, quantitative trait loci (QTL), genomic selection, gene regulatory networks, multi-omics (transcriptomics, proteomics)

## Abstract

Soybean (*Glycine max* [L.] Merr.) serves as a critical global source of plant-based protein and oil, yet the inverse relationship between seed protein content (PC) and oil content (OC) remains a major barrier to simultaneous improvement. Recent advances in genomics, transcriptomics, and proteomics have elucidated key regulatory genes and networks underlying these traits, including Gm*WRI1a*, *LEC2*, *Glyma.20G085100*, and the LAFL transcriptional module. These findings reveal that carbon and nitrogen resource partitioning during seed maturation is tightly coordinated by pleiotropic regulators, many of which mediate metabolic trade-offs that limit dual optimization. Although certain wild soybean loci and “bridge genes” like Gm*SWEET39* show potential to partially uncouple PC–OC antagonism, their effects are often context-dependent and modest in scale. This review synthesizes current understanding of the genetic architecture and metabolic frameworks that shape oil and protein accumulation in soybean seeds. It highlights promising molecular breeding strategies—including phase-specific gene regulation, CRISPR-mediated multiplex editing, and the stacking of favorable alleles—to overcome long-standing trade-offs. By leveraging multi-omics integration and functional *VAL*idation in diverse germplasm, future soybean breeding programs can more effectively develop high-protein, high-oil cultivars tailored to both nutritional and industrial demands.

## Graphical Abstract

Graphical abstract of the oil–protein trade-off in soybean seeds.A yin–yang layout summarizes genetic and regulatory factors associated with seed oil (left, light green) and seed protein (right, dark green). The oil sector highlights triacylglycerol (TAG) biosynthesis and QTLs linked to oil accumulation (e.g., signals on Chromosome 5 and 10). The protein sector shows storage-protein composition (7S/11S and their subunits) and key regulators in the LAFL network (LEC1, LEC2, FUS3, ABI3) together with hormone crosstalk (GA/ABA), bHLH TFs and AIP2, and effects on sucrose uptake and DELLA proteins. Central elements (SWEET39) and loci such as QQS, Danbaekkong (Chr20), qPro8 and qPro19 illustrate connections between both traits. Abbreviations: TAG, triacylglycerol; QTL, quantitative trait loci; GA, gibberellin; ABA, abscisic acid.

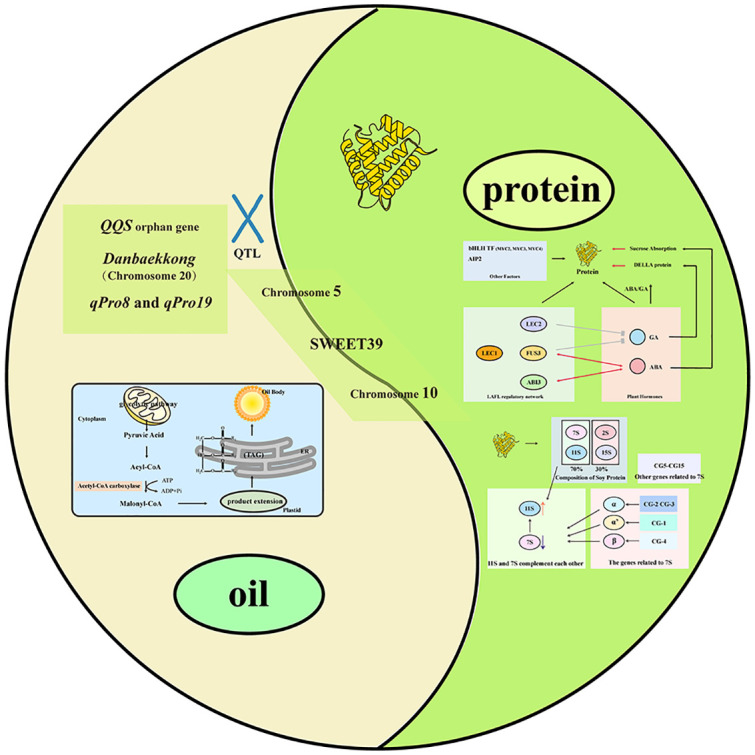

## Introduction

**Figure 1:**
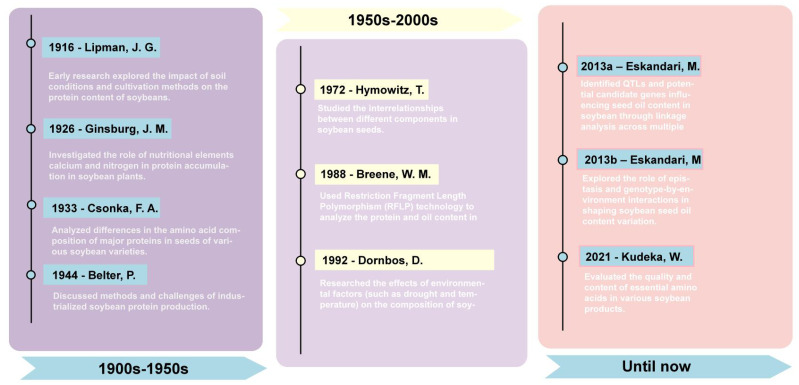
Historical timeline of soybean protein and oil research.

In recent years, the application of transcriptomics and proteomics has further advanced soybean research (Pavan Kumar et al., 2017; Fu et al., 2024; Mo et al., 2024). These technologies have helped scientists map gene expression profiles at different developmental stages and tissues of soybeans, elucidating the complex regulatory networks of soybean seed oil and protein synthesis. Through these studies, researchers have not only discovered key genes affecting soybean oil and seed protein content but also explored the expression patterns and functions of these genes under different environmental conditions (**Figure 1**). Key milestones in soybean protein and oil research: 1916 - Lipman, J. G.: Soil conditions’ impact on soybean protein (Lipman et al., 1916). 1926 - Ginsburg, J. M.: Role of calcium and nitrogen in protein accumulation (Ginsburg and Shive, 1926). 1933 - Csonka, F. A.: Amino acid composition in soybean varieties (Csonka and Jones, 1933). 1944 - Belter, P.: Industrialized soybean protein production methods (Belter et al., 1944). 1972 - Hymowitz, T.: Interrelationships in soybean seed components (Hymowitz et al., 1972). 1988 - Breene, W. M.: RFLP technology in seed protein and oil content analysis (Breene et al., 1988). 1992 - Dornbos, D.: Environmental factors’ effect on soybean composition (Dornbos and Mullen, 1992). 2013a – Eskandari, M.: Genetic control of soybean seed oil: QTL and candidate genes identification (Eskandari et al., 2013a). 2013b – Eskandari, M.: Epistatic and environmental interactions in soybean seed oil content (Eskandari et al., 2013b). 2021 - Kudelka, W.: Quality and essential amino acids in soybean products (Kudełka et al., 2021).

Soybean (*Glycine max* [L.] Merr.), as a vital food and economic crop, has its oil and seed protein content directly influencing its nutritional value, market demand, and industrial applications (Clemente and Cahoon, 2009). Globally, soybean is not only a primary source of edible oil but also a crucial plant protein raw material. Therefore, improving the seed protein and oil content has long been a central goal of breeding research (Thrane et al., 2017).

In breeding practices, the improvement of oil and seed protein content involves complex genetic backgrounds and trade-offs between traits. On the one hand, increasing seed oil content helps meet the demand for oil production; on the other hand, enhancing seed protein content is essential for food processing and the feed industry. However, these two traits are often negatively correlated, posing significant challenges for breeders, necessitating new insights from omics-based approaches (Hymowitz et al., 1972). By adopting effective breeding strategies, it is possible to enhance the economic value of soybeans, meet diverse market demands, and promote sustainable agricultural development. In the late 20th century, with the application of DNA recombinant technology, researchers began improving soybeans through genetic engineering (Rahman et al., 2023). In the 21st century, the application of high-throughput technologies such as genomics, transcriptomics, and proteomics has provided a more systematic and in-depth understanding of the molecular regulatory mechanisms of soybean oil and seed protein content. For example, through genome-wide association studies (GWAS) and quantitative trait loci (QTL) analysis, researchers have identified a series of key genes and regulatory pathways related to oil and seed protein content (Diers et al., 1992; Hwang et al., 2014; Jin et al., 2023).

This review aims to provide a comprehensive and focused summary of the genetic, genomic, transcriptomic, and proteomic insights related to soybean seed oil and seed protein content. We first discuss the current understanding of the molecular mechanisms governing protein and oil accumulation in seeds, followed by an examination of the genetic trade-offs and antagonistic relationships between these two traits. The review then highlights recent advances in genome editing technologies that offer potential solutions for simultaneous improvement of both seed protein and oil content. Finally, we present future perspectives on integrating multi-omics approaches and breeding strategies to develop high-quality soybean varieties with optimized nutritional value.

## Genetic and omics insights into soybean protein content

### Proteomics unveils key regulators of seed protein accumulation

Proteomics has become a powerful tool for elucidating protein expression dynamics and regulation during seed development in soybean. Since its inception, proteomics—the study of protein expression and function—has rapidly developed, supported by expanding plant genomic resources and EST sequence databases for protein identification (Xu et al., 2015; Islam et al., 2019; Chen et al., 2020). In recent years, proteomics has been widely applied in various aspects of soybean biology, such as growth and development, stress response, and nodulation interactions, deepening our understanding of protein expression changes throughout the soybean life cycle (Xu et al., 2015; Islam et al., 2019).

Proteomics is increasingly used to analyze enzyme expression and regulatory mechanisms during seed storage. Seed oil and seed storage proteins are the main storage materials in soybeans. Using relative and absolute quantitation isotope labeling techniques (iTRAQ), researchers identified specific proteins involved in lipid metabolism, stress response, and storage protein biosynthesis by analyzing root hair and developing seeds, thereby shedding light on the molecular mechanisms underlying seed development and nutrient accumulation (Hooker et al., 2023a; Niu et al., 2025).

The rapid advancement of proteomics, particularly with the support of LC-MS/MS technology, has provided critical insights into the regulatory mechanisms governing seed protein and oil content in soybeans. Current research hotspots focus on using proteomics to elucidate the pathways involved in the synthesis and accumulation of these key storage compounds during seed development. Studies have identified core proteins and transcription factors that play crucial roles in regulating seed protein and oil content, which are essential for breeding high-protein and high-oil soybean varieties.

Before 2012, soybean proteomics research mainly relied on two-dimensional gel electrophoresis (Xu et al., 2015). However, advancements in liquid chromatography-tandem mass spectrometry (LC-MS/MS) have enabled high-throughput proteomics, significantly improving the efficiency and accuracy of proteomics research. Subsequently, the integration of two-dimensional gel electrophoresis, semi-continuous multidimensional protein identification technology (Sec-MudPIT), and LC-MS enhanced our understanding of the metabolic processes during soybean seed filling (Niu et al., 2025).

However, challenges remain. One significant difficulty is translating these proteomic insights into practical breeding strategies that can precisely control seed protein and oil content. The complex and variable effects of environmental factors on protein and oil expression make it challenging to achieve stable and desired traits across different conditions. Additionally, effectively integrating proteomics with other omics data to fully understand the genetic and molecular basis of seed protein and oil content requires further research and technological advancements.

### Functional classification and composition of soybean storage proteins

During seed development, genes encoding storage proteins such as *β-conglycinin* and *glycinin*, as well as those involved in oil or starch synthesis, exhibit peak expression during embryo development, a period of maximum fresh weight, indicating that these storage compounds are deposited just before the drying process begins. This balance is crucial for optimizing soybean protein quality.

Soybean seeds contain three major storage components: proteins, oils, and carbohydrates, with proteins constituting approximately 40% of the dry weight in common soybean varieties (Krishnan and Jez, 2018). Protein accumulation occurs in both the embryo and the endosperm (Krishnan, 2000), and these proteins are classi*FIE*d into four components based on their sedimentation coefficients: 2S, 7S, 11S, and 15S (Singh et al., 2015). Among these, the 7S and 11S globulins are the most significant, accounting for over 70% of the total seed protein content (Ji et al., 1999), making them crucial to soybean protein composition.

**Figure 2:**
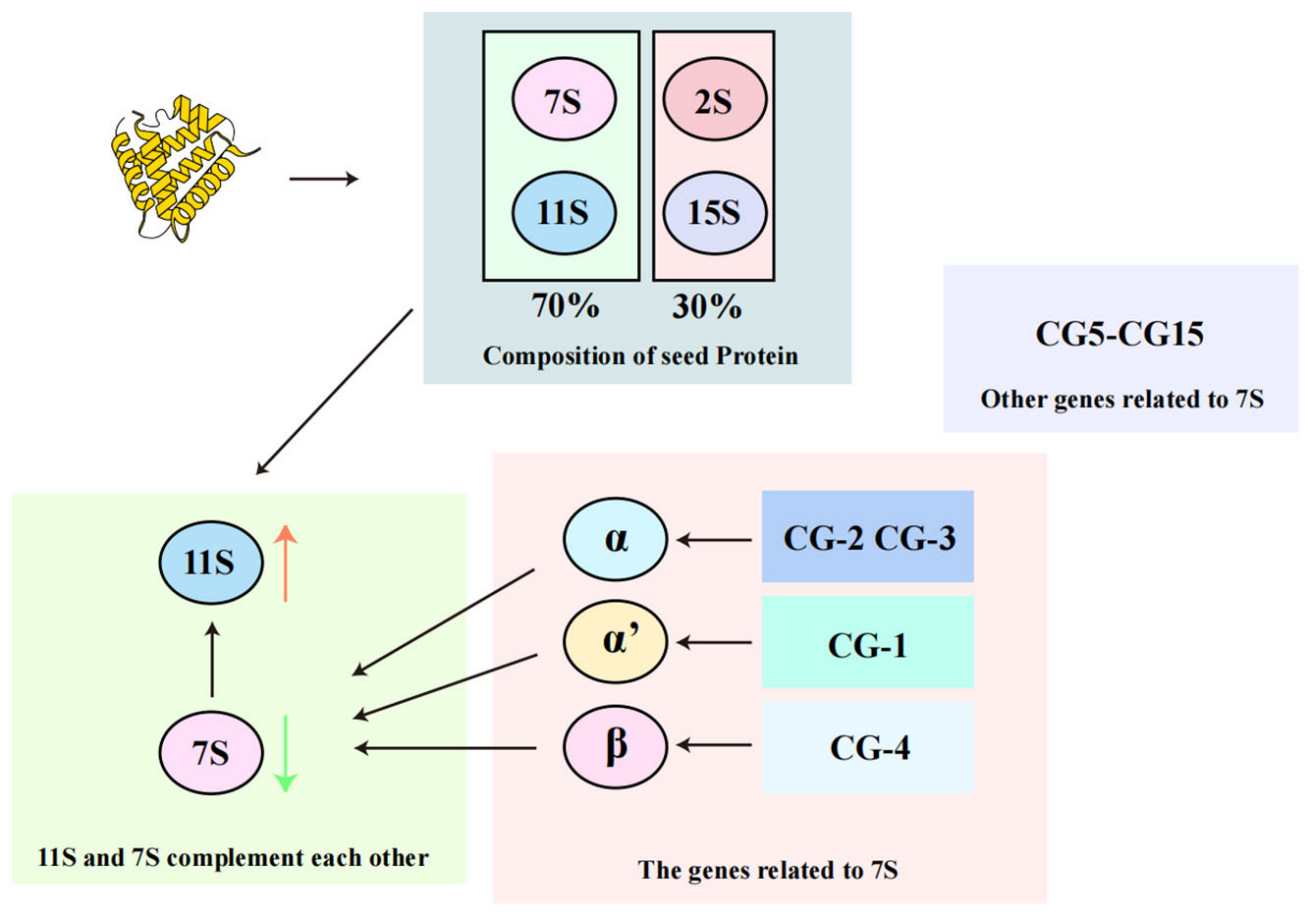
Classification of soybean proteins.

The 7S component primarily consists of *β-conglycinin*, a heterotrimer made up of *α*, *α’*, and *β subunits* encoded by 15 genes (*CG-1* to *CG-15*) (Li and Zhang, 2011; Ha and Kim, 2023) (**Figure 2**), which illustrates the classification and relative abundance of storage proteins in soybean seeds. The 11S component, which is simpler, consists only of *glycinin*, a hexamer with each subunit containing both acidic and basic polypeptides (Staswick et al., 1981; Krishnan, 2000; Singh et al., 2015). As depicted in **Figure 2**, the 11S *glycinin* is one of the two predominant storage proteins, reflecting the structural and compositional diversity of soybean seed proteins. The balance between *β-conglycinin* and *glycinin* in soybean seeds influences the amino acid profile and nutritional value of the soybean proteins (Singh et al., 2015). For instance, *glycinin* contains significantly more sulfur-containing amino acids compared to *β-conglycinin*, which is rich in lysine but has fewer disulfide bonds (Paek et al., 1997).

The classification and composition of soybean storage proteins. Soybean seeds contain four main types of storage proteins based on their sedimentation coefficients: 2S, 7S, 11S, and 15S. The 7S and 11S proteins, which include *β-conglycinin* and *glycinin* respectively, are the most abundant, together accounting for over 70% of the total seed protein content. *β-conglycinin* is a heterotrimer composed of *α*, *α’*, and *β subunits*, encoded by genes *CG-1* to *CG-15*. *glycinin*, on the other hand, is a hexamer made up of acidic and basic polypeptides, reflecting the complexity and diversity of soybean protein structure.

### Transcriptomic and co-expression analyses identify regulatory hubs

Several transcription factors have been identified as key regulators of seed development, including WRINKLED1 (*WRI1*) (Jo et al., 2024), APETALA2 (*AP2*), VIVIPAROUS1/*ABI3*-LIKE (*VAL*), FERTILIZATION INDEPENDENT ENDOSPERM (*FIE*) (Kagaya et al., 2005), GLABRA2 (*GL2*), PICKLE (*PKL*) (Santos-Mendoza et al., 2008), and DNA-binding-with-one-finger (*Dof4*) (Niñoles et al., 2022).These transcription factors influence carbohydrate, oil, and protein deposition during seed filling, and *VAL*1 and *VAL*2 are particularly critical in the transition from embryo development to germination (Gazzarrini et al., 2004).

Recently, additional hub genes such as *LEC2*, *ABI3*, and *SWEET10a* have been identified through RNA-seq and co-expression analysis (Jo et al., 2019). These datasets and hub genes provide valuable resources and candidate gene lists for functional *VAL*idation.

The identification of transcription factors such as *WRI1*, *ABI3*, and *LEC2* as central regulators of seed filling and maturation highlights a crucial hotspot in current research: understanding the precise molecular mechanisms behind these regulatory hubs.

### Hormonal and transcriptional networks in protein deposition

**Figure 3:**
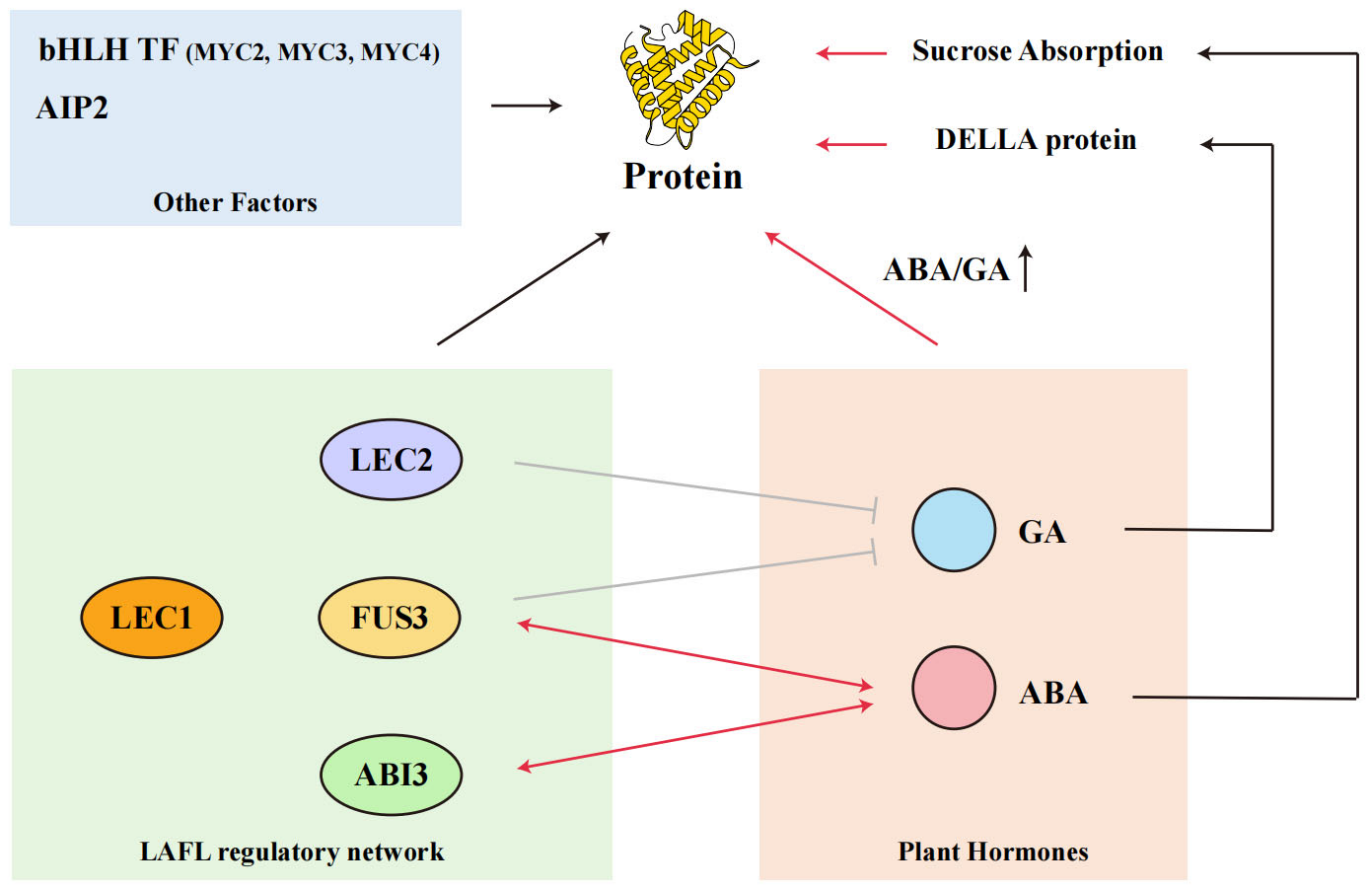
Relationship between seed protein content, transcription factors, and plant hormones GA and ABA.

Numerous transcription factors play essential roles in regulating protein accumulation in seeds. In Arabidopsis, the LAFL network—comprising *FUS3*, *ABI3*, *LEC1*, and *LEC2*—has been well-documented to control storage protein gene expression (Parcy et al., 1994; Kroj et al., 2003; Gazzarrini et al., 2004; Kagaya et al., 2005; To et al., 2006). This network is highly conserved in soybeans, suggesting a similar regulatory role, although specific functions in soybeans need further exploration (Pelletier et al., 2017). **Figure 3** highlights the intricate regulatory interactions between the LAFL network, plant hormones such as ABA and GA, and other key transcription factors involved in protein accumulation. Overexpression of Gm*LEC2a*, a key factor in this network, has been shown to enhance storage protein gene expression, underscoring the importance of transcriptional regulation in seed protein content (Manan et al., 2017). Recent research also indicates the involvement of other factors like *AIP2*, *MYC2*, and *MYC4* in this regulatory process (Gao et al., 2016; Shen et al., 2022).

Hormonal Regulation of seed protein content Plant hormones, particularly abscisic acid (ABA) and gibberellins (GA), interact closely with the LAFL network to regulate seed protein accumulation (Santos-Mendoza et al., 2008). As shown in **Figure 3**, ABA enhances storage protein accumulation during the seed filling stage by promoting the expression of storage protein genes, while the ABA/GA ratio modulates the transition between seed maturation and storage protein biosynthesis. ABA is crucial during the seed filling stage, promoting storage protein accumulation by enhancing storage capacity and inducing the expression of storage protein genes (Nonogaki; Phillips et al., 1997; Delmas et al., 2013). The ABA/GA ratio plays a critical role, with a higher ratio favoring seed maturation and storage protein accumulation (Finkelstein et al., 2002; Nambara and Marion-Poll, 2005; Leprince et al., 2017). In soybeans, ABA has been shown to increase sulfur-containing amino acids by enhancing the concentration of cysteine precursors (Kim et al., 1997). However, detailed investigations suggest that endogenous ABA concentration in soybean cotyledons correlates with rapid dry weight and sucrose accumulation during seed filling, indicating a direct physiological link between ABA and storage protein biosynthesis. Nevertheless, the molecular mechanisms underlying ABA-mediated control of protein accumulation in soybean remain to be fully elucidated (Schussler et al., 1984).

The network of transcription factors and plant hormones that influence seed protein accumulation in soybeans. Key transcription factors involved include FUSCA 3 (*FUS3*), ABA-INSENSITIVE3 (*ABI3*), LEAFY COTYLEDON1 (*LEC1*), and *LEC2*, which are crucial in regulating the expression of storage protein genes. The LAFL regulatory network, highly conserved between Arabidopsis and soybeans, plays a significant role in this process. Additionally, plant hormones such as gibberellic acid (GA) and abscisic acid (ABA) interact with these transcription factors, affecting protein accumulation. Specifically, the DELLA proteins, bHLH transcription factors (*MYC2*, *MYC3*, *MYC4*), and *AIP2* are also involved in modulating seed protein content. Understanding these interactions provides insights into the complex regulatory mechanisms governing protein synthesis in soybean seeds.

### QTL mapping and molecular targets for protein trait improvement

**Figure 4:**
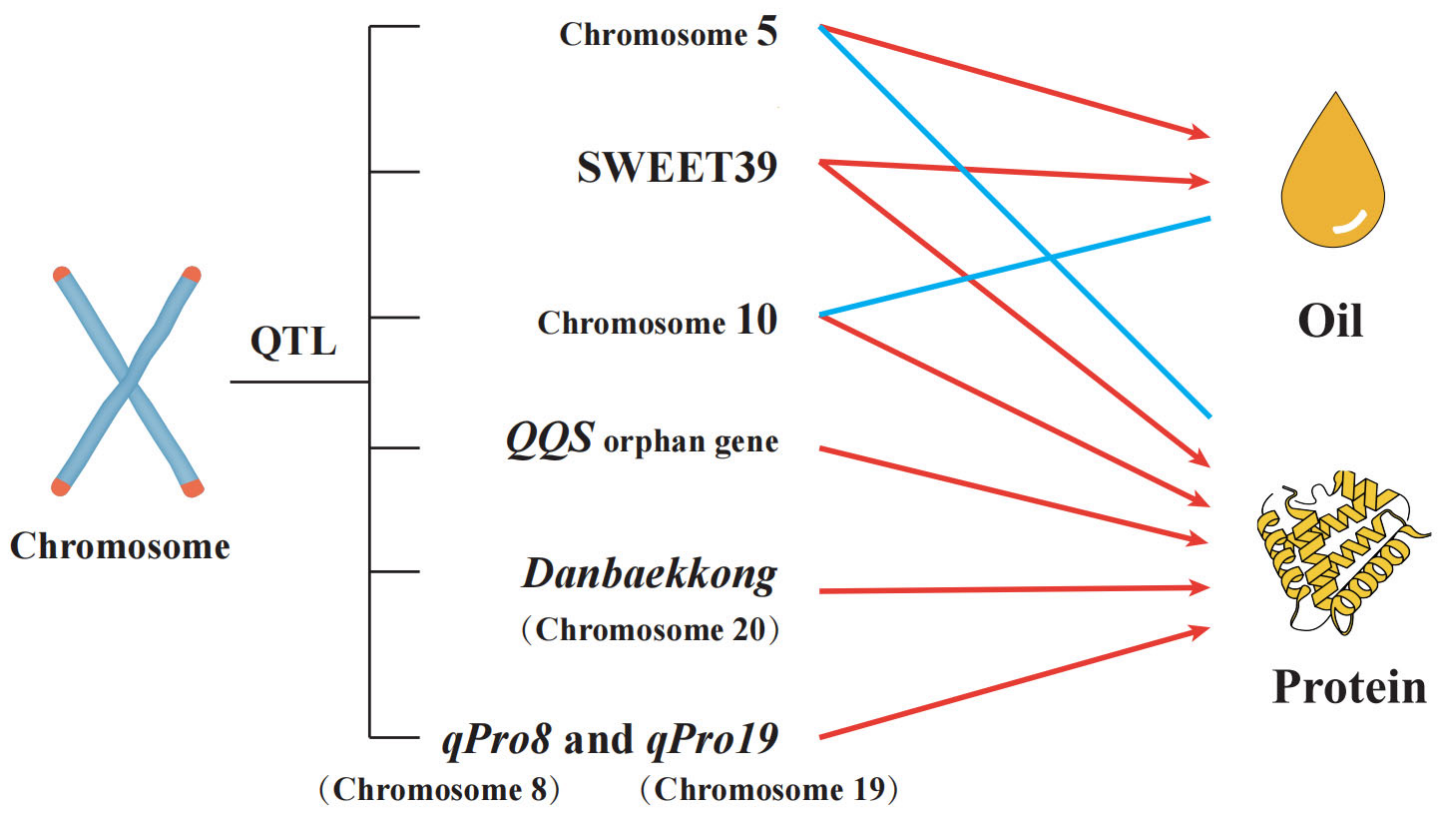
Impact of QTL on seed Oil and protein content.

QTL Mapping and Genetic Studies Significant progress has been made in identifying QTLs associated with seed protein content in soybeans (**Figure 4**). For example, LG I on chromosome 20 has been consistently linked to high-protein alleles, with recent studies confirming *Glyma.20G085100* as a candidate gene that can increase seed protein content by 2%-3% (Fliege et al., 2022). Advanced QTL mapping and GWAS have also identified new loci, such as those associated with the *QQS* gene, which influences carbon and nitrogen allocation (Li et al., 2015; Kumar et al., 2023; Li et al., 2023). Additionally, several studies have identified meta-QTLs on chromosomes 6 and 20 that are promising targets for improving both oil and seed protein content (Kumar et al., 2023). Emerging Areas and Challenges While much has been learned about the genetic regulation of seed protein content, challenges remain in integrating this knowledge into practical breeding programs. The pleiotropic effects of key genes, such as Gm*SWEET39*, which affects both seed protein and oil content, complicate breeding efforts aimed at simultaneously optimizing these traits (Zhang H. et al., 2020). Furthermore, the role of lncRNAs, such as lncRNA43234, in regulating seed protein content represents an emerging area of research that could provide new avenues for enhancing soybean protein quality (Zhang et al., 2023). However, fully elucidating the complex network of genes and pathways involved in protein accumulation remains a significant challenge.

This figure illustrates the quantitative trait loci (QTL) affecting oil and seed protein content in soybeans. Notable QTLs include the “Danbaekkong” (Dan) protein allele on chromosome 20, which is associated with higher protein concentrations but lower seed oil content and yield. Studies utilizing marker-assisted selection (MAS) have shown that lines with the Dan protein allele exhibit these traits. Additionally, the *QQS* orphan gene, which influences carbon and nitrogen allocation through interaction with the *NF-YC* transcription factor, has been shown to increase seed protein content when overexpressed. QTL mapping identified key regions on chromosomes 5, 8, 10, and 19, with significant contributions to seed oil content variation. For example, a QTL localized on chromosome 8 includes the gene *SWEET39*, and chromosome 19 includes *qPro19*, both impacting protein and oil accumulation. This comprehensive mapping helps to uncover the genetic mechanisms governing seed oil and seed protein content, providing valuable targets for soybean breeding programs.

## Genetic and omics insights into soybean oil content

### Transcriptomic and proteomic insights into oil synthesis

Transcriptomics and functional genomics studies have revealed that mutations in genes such as *FAD2-1A* and *FAB2C* significantly alter oleic and stearic acid levels in soybean seeds (Ma et al., 2021). Additionally, the discovery of gene networks and mutations influencing fatty acid composition, such as those in *FAD2-1A*, underscores the growing importance of metabolic engineering in crop improvement. Islam et al. (2019) performed quantitative proteomics analysis of low-linolenic acid transgenic and control soybean seeds, revealing disturbances in protein abundance related to fatty acid metabolism pathways, including reductions in proteins associated with FA initiation, elongation, desaturation, and *α*-linolenic acid *β*-oxidation (Islam et al., 2019). Xu et al. (2015) analyzed high-oil transgenic soybeans with overexpressed *GmDGAT1–2* using quantitative proteomics and lipidomics, demonstrating that overexpression of *GmDGAT1–2* led to downregulation of peroxidases and upregulation of oleic acid, significantly altering the total fatty acid composition (Xu et al., 2015; Chen et al., 2020). In a complementary study, Torabi et al. (2021) showed that downregulation of three DGAT1 genes resulted in reduced oil content but increased protein and oleic acid levels, highlighting the dual regulatory role of DGAT1 in seed composition (Torabi et al., 2021). In addition to *GmDGAT1-2*, other genes such as *GmDGAT3-2*, which exhibits high specificity for oleic acid (C18:1), and *GmWRI1c*, a natural variant affecting palmitic acid levels, have emerged as key metabolic regulators. Omics integration further highlights *GmGPAT* and Gm*GAPDH* as potential metabolic bottlenecks connecting glycolysis with TAG biosynthesis.

### Transcriptional regulation of oil biosynthesis

**Figure 5:**
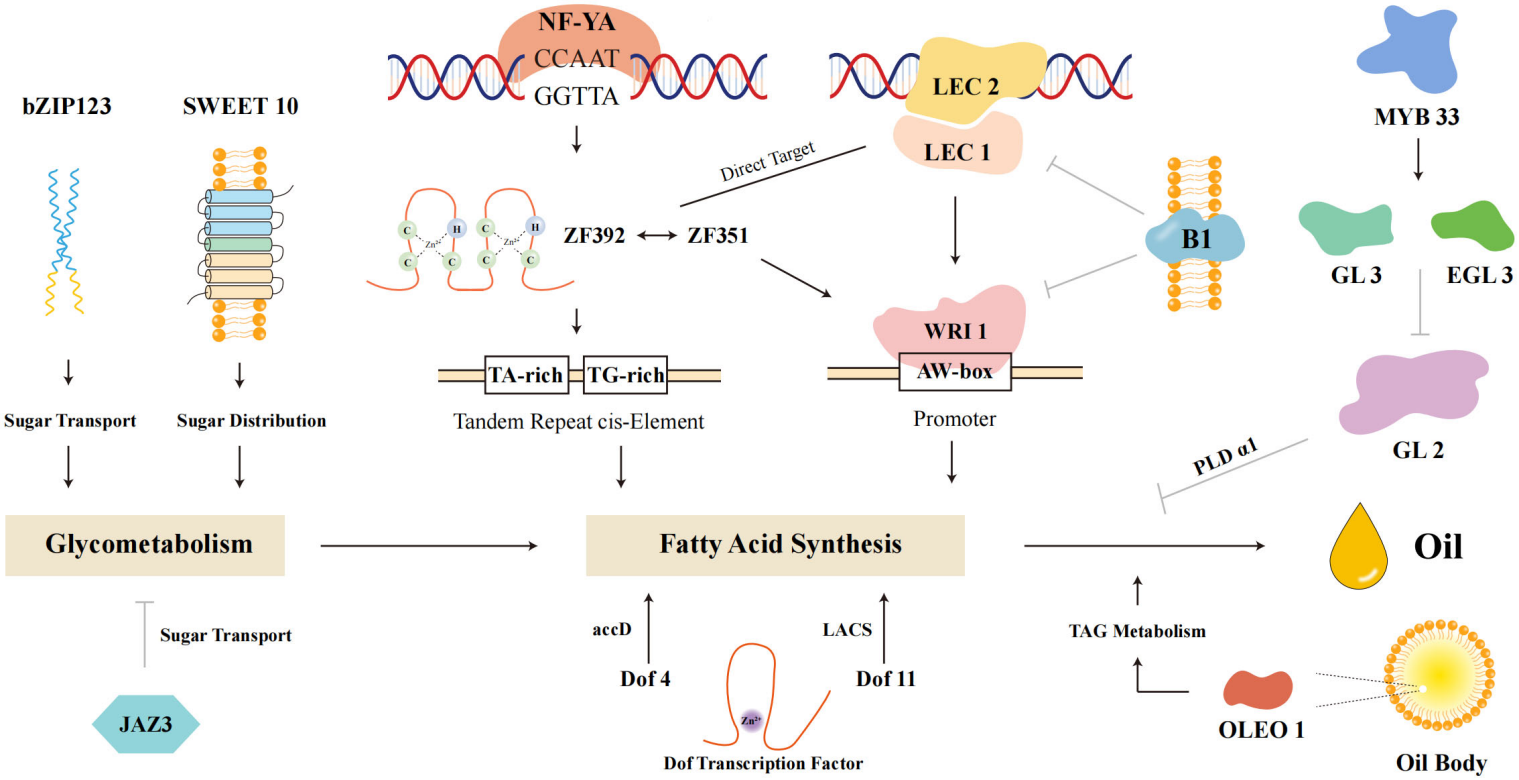
Genetic network of oil synthesis in soybeans.

Several transcription factors have been identified as key regulators of oil biosynthesis in soybeans. Gm*LEC2*, a B3 domain transcription factor, enhances TAG content by activating lipid biosynthesis genes (Manan et al., 2017). As shown in **Figure 5**, Gm*LEC2* is part of the intricate genetic network regulating oil biosynthesis, interacting with other transcription factors such as *NF-YA*, *LEC1*, and *Dof* proteins to modulate key metabolic pathways. Gm*WRI1a*, under both constitutive and seed-specific promoters, significantly increases seed oil content by activating genes involved in various steps of lipid accumulation (Chen et al., 2018; Wang et al., 2022). *SWEET10*, a sugar transporter gene, has been shown in soybean that Gm*SWEET10a* and Gm*SWEET10b* mediate sucrose and hexose transport from seed coat to embryo, impacting seed size and composition (Wang et al., 2020). While direct regulation by Gm*WRI1a* in soybean has not yet been confirmed, *SWEET10* is considered a potential contributor to the carbon supply required for fatty acid and TAG biosynthesis. The synergistic action of Gm*ZF351* and Gm*ZF392* further boosts lipid biosynthesis, with both factors being direct targets of Gm*LEC1* (Li et al., 2017). These transcription factors are integrated within a complex regulatory network that includes tandem repeat cis-elements and promoters, enabling fine-tuned modulation of oil synthesis pathways.

Complex genetic network involved in oil synthesis within soybeans. Key components include fatty acid synthesis pathways, sugar transport mechanisms, and the regulation of oil body formation. Transcription factors such as *NF-YA*, *LEC1*, *LEC2*, and *Dof* proteins play crucial roles in regulating genes involved in these pathways. The network highlights the interplay between various genetic elements, including tandem repeat cis-elements and promoters, which modulate the expression of genes like *SWEET10* and *WRI1* that are essential for glycometabolism and TAG metabolism. Understanding these interactions provides insights into the regulation of seed oil content and offers potential targets for genetic enhancement to improve soybean oil yield.

### Fatty acid accumulation and synthesis pathways

**Figure 6:**
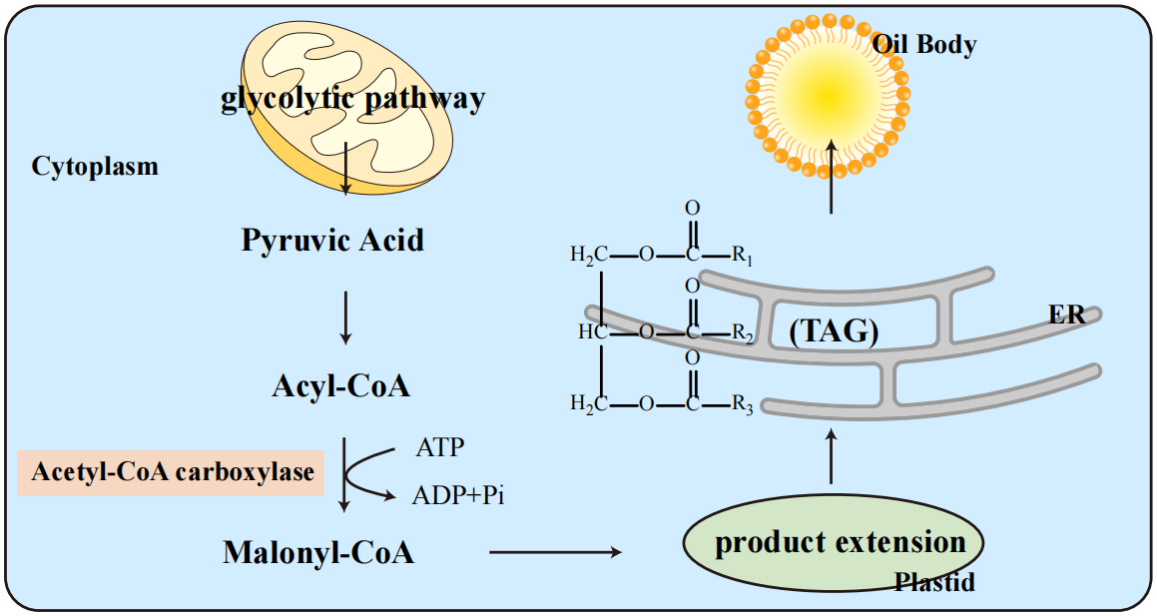
Metabolic pathway of oil body formation in soybeans.

The fatty acid (FA) accumulation cycle in soybeans is relatively short, typically occurring in the cotyledons. In plant seeds, FAs are primarily stored as triacylglycerol (TAG), whose biosynthesis depends on carbohydrate flux, such as sucrose from photoautotrophic tissues (Allen et al., 2009). **Figure 6** illustrates the metabolic pathway of oil body formation in soybeans, beginning with glycolysis in the cytoplasm and progressing through the key intermediates, including acetyl-CoA and malonyl-CoA, which are crucial for triacylglycerol (TAG) synthesis in the endoplasmic reticulum. The process begins with the *de novo* synthesis of FA from acetyl-CoA, catalyzed by acetyl-CoA carboxylase, the rate-limiting enzyme in FA synthesis (Sasaki and Nagano, 2004). The FA synthase complex then elongates these FAs in plastids before they are exported to the endoplasmic reticulum to form TAG (Ohlrogge and Browse, 1995; Rawsthorne, 2002). These TAGs are stored in oil bodies, surrounded by a phospholipid monolayer embedded with proteins (Tzen et al., 1993; Napier et al., 1996).

The metabolic processes involved in the synthesis of oil bodies in soybeans. The process begins with glycolysis in the cytoplasm, where pyruvic acid is converted into acetyl-CoA. This acetyl-CoA is then carboxylated by acetyl-CoA carboxylase to form malonyl-CoA in the plastid. The subsequent steps involve the extension of carbon chains and the synthesis of triacylglycerols (TAGs) in the endoplasmic reticulum (ER). The TAGs are eventually packaged into oil bodies. This metabolic pathway highlights the crucial steps and key intermediates such as acyl-CoA and malonyl-CoA, which are essential for the production and storage of oils in soybean cells.

### Genetic regulation of soybean seed oil content

Refer to **Figure 6** to illustrate the biochemical context of how key genes and regulatory elements impact oil body formation. Soybean oil is composed mainly of five fatty acids: palmitic acid, stearic acid, oleic acid, linoleic acid, and linolenic acid. Genetic mutations in key genes such as Gm*KASIIB*, Gm*FATB*, and Gm*FATA* have been shown to significantly alter the composition of these FAs (Ma et al., 2021; Devereaux et al., 2024; Liao et al., 2024). For example, mutations in Gm*FATB* can reduce palmitic acid content, contributing to lower saturated FA levels (Ma et al., 2021). Conversely, overexpression of Gm*FATA* enhances overall FA biosynthesis and may increase seed oil yield (Zhou et al., 2021). Gm*PDCT* mediates phosphatidylcholine-diacylglycerol interconversion, influencing TAG assembly, while *SDP1* is involved in lipid catabolism; mutations in these genes can alter oil body stability and overall oil content (Aznar-Moreno et al., 2022; Wei et al., 2025). The role of *FAD2* and *FAD3* in converting oleic acid to linoleic and linolenic acids, respectively, has been well-documented, with targeted mutations resulting in increased oleic acid content (Okuley et al., 1994; Haun et al., 2014; Lakhssassi et al., 2017; al Amin et al., 2019; Torabi et al., 2021). Additionally, targeted mutations in genes such as Gm*PDCT* and *SDP1* have been demonstrated to significantly alter fatty acid composition in soybean seeds. Recent studies using CRISPR/Cas9-mediated knockout of Gm*PDCT1* and Gm*PDCT2* revealed that disruption of these genes increases the proportion of monounsaturated fatty acids (MUFAs) while reducing polyunsaturated fatty acids (PUFAs), thereby improving oil quality and offering a viable strategy to increase total seed oil content. Similarly, mutation of *SDP1*, which is involved in TAG catabolism, enhances oil accumulation and stability during seed maturation (Graham, 2008; Kanai et al., 2019; Ulch et al., 2025).

### QTL and GWAS discoveries for oil content

Recent studies using QTL and GWAS analyses have identified several loci and candidate genes associated with seed oil content in soybeans. QTLs associated with seed oil content have been repeatedly mapped to chromosomes 8, 10, and 20, often explaining 5-15% phenotypic variance, for example, in the NJMN-RIL linkage population, *qOil-5–1* and *qOil-10–1* explained 6.3-26.3% variance; in multiple-environment GWAS, oil QTLs on chromosomes 8 and 20 accounted for up to ~14.9% variance (Cao et al., 2017; Gillenwater et al., 2022). For instance, Gm*MFT*, which regulates embryo size and development, has been positively correlated with triacylglycerol (TAG) accumulation, and Gm*OLEO1*, encoding an oil body-associated protein, has been shown to increase seed oil content by up to 10% in transgenic lines (Zhang et al., 2019; Cai et al., 2023). The integration of these findings into breeding programs has the potential to develop high-oil soybean varieties, while further exploration of genes like Gm*DREBL* and Gm*B1* may offer additional strategies for manipulating seed oil content (Zhang et al., 2016; Zhang et al., 2018).

## Trade-offs between oil and protein in soybeans

One of the most significant challenges in soybean breeding is achieving an optimal balance between oil and seed protein content, given the often inverse relationship between these two traits. This trade-off arises in part from shared biosynthetic pathways and competition for carbon and nitrogen resources during seed development (Patil et al., 2017; Kambhampati et al., 2019). Historically, breeding programs that prioritized one trait often observed a corresponding decline in the other, making simultaneous improvement particularly difficult development. Nevertheless, both traits are of critical importance. High oil content is valuable for edible oil production and industrial applications, while high protein content is essential for animal feed and plant-based protein markets (Singer et al., 2023; Wu et al., 2024). One of the key goals in modern soybean breeding is to enhance seed oil content without compromising other essential traits such as yield or protein quality. Conversely, there are scenarios where reducing seed oil content in soybeans is desirable, such as in the production of soybeans specifically bred for high seed protein content. Moving forward, breeders must strike a balance between optimizing oil and seed protein content and maintaining other essential agronomic traits, such as yield and disease resistance. Achieving this requires a nuanced understanding of the genetic, biochemical, and physiological basis of these traits, as well as innovative breeding approaches.

In fact, both oil and protein are in high demand in the soybean industry, particularly in the food and livestock sectors. Breeding varieties with either high oil or high protein content can serve specific market niches, but developing soybean cultivars with both high oil and protein levels offers more flexibility and greater value in localized supply chains. A singular focus on either oil or protein may be overly simplistic. Instead, considering the total nutritional yield—for instance, a variety that produces x grams of oil and y grams of protein per unit area—offers a more comprehensive assessment. By applying a simple additive model, such as x + y (or a weighted index), breeders can more effectively identify genotypes that maximize total nutritional productivity. This integrated approach holds promise for advancing soybean breeding goals in a more holistic and practical manner.

Seed protein content (PC) and oil content (OC) typically exhibit an inverse correlation in soybean due to the competition for limited carbon and nitrogen resources during seed maturation, consistent with classical C–N partitioning hypotheses (Hooker et al., 2023b; Niu et al., 2025). Recent genome-wide association studies have revealed widespread QTL overlaps between PC and OC, and Diers et al. (2023) further confirmed that the vast majority (94%) of trait-associated loci have antagonistic effects on protein and oil content, reinforcing the complexity of this genetic trade-off (Diers et al., 2023; Jin et al., 2023). Recent transcriptome and co-expression network analyses further demonstrate that during late seed maturation, high-oil cultivars preferentially activate lipid-centric pathways, while high-protein cultivars shift toward nitrogen-assimilation and protein biosynthesis modules, mediated by distinct core regulators (e.g. PEBP-CLO1 vs GS-PTR1) (Niu et al., 2025; Patel et al., 2025). Integrating knowledge of pleiotropic regulators (e.g. GmSWEET39, LEC2, ABI3) with network−level understanding offers a promising route for developing soybean varieties that balance PC and OC rather than compromise one for the other.

Building on the classical C–N partitioning framework, the gene sets discussed above clearly illustrate why the negative correlation between seed protein content (PC) and oil content (OC) remains so challenging to break. Many of the genes that strongly enhance OC—such as GmWRI1a, GmLEC2, FATA/FATB, OLEO1, MFT, and PDCT/SDP1—act primarily by boosting carbon flux toward fatty-acid and triacylglycerol (TAG) biosynthesis, which can constrain resources available for storage-protein deposition. Conversely, protein-associated regulators such as the CG gene family (β-conglycinin subunits), Glyma.20G085100, QQS–NF-YC modules, and LAFL network members (LEC1, ABI3, FUS3, LEC2a) tend to favor nitrogen assimilation and storage-protein accumulation, often with an accompanying reduction in oil. This antagonism is further reinforced by overlap of QTL for PC and OC on key chromosomes (e.g., 6, 8, 10, and 20), where alleles that increase one trait frequently decrease the other. Genes capable of increasing one trait without depressing the other remain scarce within cultivated backgrounds. For example, Glyma.20G085100 has been associated with higher PC (≈2–3%) and shows limited pleiotropic penalties in some contexts, though its effect may vary with genetic background and environment. Similarly, wild soybean–derived loci provide promising leads to partially uncouple PC–OC antagonism, but the effect sizes are often modest and environment-dependent. Pleiotropic regulators that connect carbon allocation, transport, and seed-filling programs—such as GmSWEET39 together with master regulators including LEC2 and ABI3—represent attractive “bridge nodes,” yet their network-level consequences for simultaneous PC–OC optimization are not fully resolved. Collectively, these observations define a central bottleneck for breeding: identifying or engineering regulators that (i) improve PC and OC concurrently or (ii) buffer the negative impact on one trait when the other is enhanced. Addressing this bottleneck will require network-level strategies that integrate multi-omics datasets, exploit allelic diversity from wild germplasm, and dissect the dynamic regulation of shared nodes during seed maturation.

High-protein strategies typically focus on enhancing the accumulation of 11S globulins, particularly glycinin, due to its higher content of essential sulfur-containing amino acids. Approaches such as silencing genes involved in 7S globulin (β-conglycinin) synthesis or overexpressing transcription factors like GmLEC2a that promote 11S protein accumulation are key strategies for boosting seed protein content. Additionally, leveraging QTLs associated with high-protein alleles, such as those on chromosome 20, can further enhance protein levels.

For example, downregulating the LAFL network or modifying the ABA/GA hormonal balance could lead to reduced storage protein accumulation.

These strategies provide a framework for manipulating soybean seed protein content to meet specific breeding objectives, whether for nutritional enhancement, optimizing processing qualities, or balancing seed protein and oil content in soybean varieties.

This section provides a comprehensive exploration of the genetic regulatory network governing seed oil content in soybeans, focusing on recent advances in fatty acid synthesis, accumulation, and the underlying regulatory mechanisms. Key highlights include the detailed dissection of fatty acid synthesis pathways and the roles of critical genes such as GmKASIIB, GmFATB, and GmFATA. Mutations and regulatory modifications of these genes have been shown to significantly impact the fatty acid composition in soybean seeds. A major hotspot in this research is the use of transcriptional regulators like GmLEC2, GmWRI1a, and the synergistic action of GmZF351 and GmZF392 to enhance seed oil content. Additionally, GWAS and QTL analyses have identified new loci and candidate genes associated with seed oil content, offering valuable resources for soybean breeding programs.

This section provides an in-depth exploration of the genetic regulatory network governing seed oil content in soybeans, with a particular focus on recent advances in fatty acid synthesis, accumulation, and the underlying regulatory mechanisms. By dissecting the fatty acid synthesis pathways and key genes such as GmKASIIB, GmFATB, and GmFATA, the text reveals how mutations and regulatory modifications in these genes can significantly impact the fatty acid composition in soybean seeds. These findings offer valuable genetic resources for breeding programs, particularly in the application of transcriptional regulators like GmLEC2, GmWRI1a, and the synergistic action of GmZF351 and GmZF392 to enhance seed oil content. Breeding strategies for high seed oil content include the overexpression of key genes such as GmFATA and GmWRI1a, suppression of FAD2 and FAD3 to increase oleic acid content, and leveraging QTL and GWAS-identified genes like GmMFT and GmOLEO1 to boost oil accumulation. On the other hand, strategies for low seed oil content focus on mutating the GmFATB gene to reduce saturated fatty acid content, downregulating oil synthesis-related transcription factors like GmWRI1a and GmLEC2, and regulating genes such as GmPDCT and SDP1 to decrease TAG accumulation. These strategies provide tailored approaches to breeding, enabling the precise manipulation of seed oil content in soybeans to meet diverse market demands.

However, significant challenges remain. Despite these insights into the genetic and molecular mechanisms, integrating this knowledge into practical breeding strategies that deliver consistent improvements in seed oil content across different environmental conditions is difficult. Furthermore, many of these findings are based on homologs identified in model plants like Arabidopsis, which raises the need for further research to validate these mechanisms in soybeans. Future research must delve deeper into the unique aspects of soybean biology to develop more precise and effective breeding strategies.

## Genome editing and molecular breeding strategies for breaking the oil–protein trade-off

Achieving simultaneous improvement in soybean oil and seed protein content demands a system-level rethinking of metabolic architecture. Instead of treating these traits as isolated targets, future strategies should embrace a modular optimization framework—identifying bottlenecks within distinct biochemical and regulatory layers, and prioritizing precise edits that realign carbon and nitrogen allocation toward dual accumulation. We propose a layered design that includes: (i) precursor supply (sucrose, amino acid transport), (ii) flux direction (key transcription factors and metabolic enzymes), (iii) storage efficiency (protein/oil body assembly), and (iv) metabolic stability (feedback inhibition, turnover prevention).

At the precursor level, sucrose and amino acid transporters serve as gateways that determine substrate availability. Genes such as *SWEET10a* and Gm*SWEET39* facilitate carbon influx into developing seeds, with Gm*SWEET39* exhibiting pleiotropic control over both oil and protein partitioning (Zhang J. et al., 2020). Enhanced sugar influx, coupled with Gm*GAPDH*−mediated glycolytic acceleration, ensures a robust carbon backbone for downstream lipid synthesis (Xu et al., 2015; Chen et al., 2020). On the nitrogen side, genes like GS and PTR1 have been linked to amino acid precursor flow, especially under protein-oriented metabolic states (Patel et al., 2025), although direct functional confirmation in soybean is pending.

Metabolic flux control is predominantly governed by a small set of master regulators. On the lipid side, Gm*WRI1a*, Gm*LEC2*, *FATA*/*FATB*, *PDCT*, and DGAT1–2 constitute a high-efficiency axis for fatty acid synthesis and triacylglycerol (TAG) packaging (Xu et al., 2015; Manan et al., 2017; Chen et al., 2018; Guo et al., 2020; Ma et al., 2021; Zhou et al., 2021; Aznar-Moreno et al., 2022; Wang et al., 2022; Devereaux et al., 2024; Liao et al., 2024; Wei et al., 2025). In contrast, protein accumulation is driven by *CG-1*~*CG-15* (β−con*glycinin* subunits), *Glyma.20G085100*, and components of the LAFL network—particularly *LEC1*, *LEC2a*, *ABI3*, and *FUS3*—which regulate storage protein gene expression in coordination with hormonal cues such as ABA/GA ratios (Nonogaki; Parcy et al., 1994; Phillips et al., 1997; Finkelstein et al., 2002; Kroj et al., 2003; Gazzarrini et al., 2004; Kagaya et al., 2005; Nambara and Marion-Poll, 2005; To et al., 2006; Santos-Mendoza et al., 2008; Li and Zhang, 2011; Delmas et al., 2013; Leprince et al., 2017; Manan et al., 2017; Ha and Kim, 2023). These modules may be independently upregulated using tissue−specific promoters to minimize metabolic competition. Furthermore, *SDP1*, a lipase responsible for TAG degradation, can be suppressed to prevent unnecessary carbon loss during seed maturation (Wei et al., 2025).

Storage stability is another overlooked but critical factor. Oil bodies formed via Gm*OLEO1* and SEIPINs ensure lipid retention and seed quality (Zhang et al., 2019; Cai et al., 2023). Protein stability, in turn, may benefit from enhancing sulfur-rich subunits (e.g., *glycinin*) and fine-tuning post-translational folding chaperones (Paek et al., 1997). Additionally, recent studies show that lncRNA43234, *AIP2*, and *MYC2*/4 modulate oil–protein partitioning by interfacing with core hormonal and developmental pathways (Gao et al., 2016; Shen et al., 2022; Zhang et al., 2023), though functional *VAL*idation in soybean remains preliminary.

Collectively, these components define a flexible yet integrated engineering blueprint: high-efficiency carbon sinks (TAG + storage protein), protected from turnover, supported by maximal precursor influx, and coordinated via pleiotropic regulators (e.g., Gm*SWEET39*, *LEC2*, *ABI3*). This sets the stage for a synthetic assembly of “elite trait modules”.

To practically overcome the protein–oil trade-off, precise combinations of gene edits targeting distinct metabolic modules offer a promising way forward. For example, simultaneous overexpression of Gm*WRI1a* and Gm*LEC2* under a seed-specific promoter could enhance fatty acid biosynthesis without altering vegetative growth, while RNAi-mediated knockdown of *SDP1* may further prevent post-maturation lipid degradation. On the protein side, activating *Glyma.20G085100* and overexpressing *CG-1* to *CG-15* subunits—especially those favoring sulfur-rich amino acids—could boost protein content and quality. Importantly, including bridge regulators such as Gm*SWEET39*, whose dual role in sugar transport and signaling impacts both oil and protein pathways, offers an avenue to coordinate both traits simultaneously. In a more advanced design, combining *FATA*/*FATB* variants with edited *QQS*–*NF-YC* regulatory modules may improve both carbon and nitrogen partitioning efficiency. These multilayered combinations, tested across diverse genetic backgrounds and environments, represent tangible steps toward engineering soybean lines that approach the theoretical maximum of x + y yield potential.

A promising but underutilized strategy involves phase-specific regulation of key metabolic modules to temporally decouple oil and protein accumulation. For instance, by driving fatty acid biosynthesis genes (e.g., Gm*FATA*, *WRI1*, DGAT1-2) during mid-seed maturation, followed by late-stage activation of protein-related regulators (*Glyma.20G085100*, CG genes, or *ABI3*), breeders can exploit the natural developmental sequence of storage compound deposition. This design aligns with observed transcriptomic data showing that TAG biosynthesis peaks earlier, while protein synthesis extends into late seed fill. Moreover, phase-tuned promoters (such as those responsive to ABA or sugar signals) may allow for non-overlapping optimization windows, reducing resource conflict. Such a “temporal separation” strategy offers a novel route to circumvent trade-offs by scheduling rather than combining resource allocation events.

## Future prospects

Despite significant progress in understanding the genetic and molecular underpinnings of seed oil and protein content in soybean, breaking the intrinsic trade-off between these traits remains a formidable challenge. As highlighted throughout this review, this antagonism arises from deeply embedded metabolic conflicts—primarily carbon–nitrogen competition and overlapping transcriptional regulation—that limit the potential for synchronous trait enhancement (Patil et al., 2017; Kambhampati et al., 2019; Diers et al., 2023; Patel et al., 2025).

Although soybean is a self-pollinating crop, early breeding efforts have explored the use of recurrent selection to improve seed oil concentration. For example, Burton & Brim (1981) demonstrated that multiple cycles of selection for oil content could gradually raise oil levels—albeit with some decrease in protein content (Burton and Brim, 1981). Similarly, Nguyen et al. (2001) applied recurrent selection to soybean lines regenerated from tissue cultures and observed modest increases in seed oil content, though accompanied by reduced protein and yield (Nguyen et al., 2001). These findings highlight the potential—but also the limitations—of recurrent selection in soybean oil improvement. Modern approaches such as marker-assisted recurrent selection (MARS) or genomic recurrent selection (GRS) may overcome some of these challenges by tracking favorable alleles more efficiently.

Looking ahead, future efforts must focus on integrating multi-layered strategies that simultaneously address metabolic flux, regulatory control, and environmental adaptability. First, precision engineering of gene combinations, such as concurrent activation of Gm*WRI1a* and *LEC2* for oil biosynthesis (Manan et al., 2017; Wang et al., 2022), with *Glyma.20G085100* and *CG* genes for protein enhancement (Li and Zhang, 2011; Fliege et al., 2022; Ha and Kim, 2023), offers a direct path toward stacking favorable alleles. Suppressing negative regulators such as *SDP1 (* Wei et al., 2025), and incorporating “bridge” genes like Gm*SWEET39* and *QQS*–*NF-YC* modules (Li et al., 2015; Zhang J. et al., 2020; Li et al., 2023), may further balance carbon and nitrogen allocation without strong pleiotropic penalties.

Second, temporal separation strategies—such as mid-maturation activation of lipid pathways followed by late-stage expression of protein-related modules—hold promise for optimizing both traits within a single developmental timeline. The use of phase-specific promoters or ABA/sugar-responsive regulatory elements (Nonogaki; Phillips et al., 1997; Finkelstein et al., 2002; Nambara and Marion-Poll, 2005; Delmas et al., 2013; Leprince et al., 2017) could further refine these efforts, leveraging the natural dynamics of seed filling (Mu et al., 2008; Niu et al., 2025; Patel et al., 2025).

Third, network-level modeling and genotype-by-environment interaction analysis will be essential for translating laboratory gains into field stability. Integrating multi-omics datasets (e.g., transcriptome, proteome, metabolome) with machine learning frameworks may help predict optimal trait combinations under diverse growing conditions (Baud et al., 2007; Xu et al., 2015; Manan et al., 2017; Pavan Kumar et al., 2017; Chen et al., 2020; Fu et al., 2024; Mo et al., 2024).

Finally, leveraging wild soybean allelic diversity (Diers et al., 2023; Jin et al., 2023), combined with marker-assisted backcrossing and CRISPR-mediated fine-tuning (Zhang J. et al., 2020; Rahman et al., 2023), can introduce novel variants capable of partially uncoupling oil–protein antagonism. The continued accumulation of functional genomic data, high-quality reference genomes, and precise phenotyping tools will empower a new era of rational soybean design.

In conclusion, breaking the oil–protein trade-off is no longer a theoretical ambition but a practical breeding objective. By combining system-level insights, advanced genetic engineering, and strategic trait scheduling, future soybean varieties may overcome historical limitations and approach the dual ideal of high oil and high protein productivity.
